# Siltuximab-Related Favorable Clinical Outcome for a Patient Suffering from Idiopathic Multicentric Castleman Disease

**DOI:** 10.1155/2022/1840589

**Published:** 2022-03-30

**Authors:** Stamatios Chrysochoou, Andreas Kreft, Eberhard Schneider

**Affiliations:** ^1^GPR Gesundheits- und Pflegezentrum Rüsselsheim Gemeinnützige GmbH, August-Bebel-Straße 59, Rüsselsheim Am Main 65428, Germany; ^2^Institute of Pathology, Johannes Gutenberg University, Langenbeckstraße 1, Mainz 55131, Germany; ^3^Department of Biology, Julius Maximilians University, Am Hubland, Würzburg 97074, Germany

## Abstract

*Rational*Castleman disease is a rare lymphoproliferative disorder that can be subdivided into unicentric and multicentric forms, the latter of which causes a spectrum of serious medical conditions. Here, we present a case of idiopathic multicentric Castleman disease in the eighth decade of life. *Patient Concerns*. First hospitalized due to unexplained progressive anemia, the patient was readmitted to the hospital 18 months later with suspected lymphoma. Clinical examination revealed a progressive lymphadenopathy. *Diagnoses*. Histopathologic lymph node features, anemia, elevated CRP and IL6 levels, splenomegaly, and hypoalbuminemia indicated multicentric Castleman (MCD) disease. *Interventions*. The patient was treated intravenously with a dose of 11 mg/kg siltuximab every 3 weeks. *Outcomes*. Timely correct diagnosis through the stringent use of consensus diagnostic criteria and sufficient siltuximab therapy has considerably promoted favorable clinical outcomes in a patient suffering from MCD.

## 1. Introduction

Castleman disease (CD) is a rare lymphoproliferative disorder first reported by pathologist Benjamin Castleman in 1954 [1]. CD is subdivided into unicentric CD (UCD), involving just one enlarged lymph node, and multicentric CD (MCD), affecting multiple lymph nodes [2]. While UCD is considered a benign condition, MCD is characterized by a variety of clinical and laboratory abnormalities with a poor prognosis in otherwise untreated patients [3]. MCD can be caused by human herpesvirus-8 (HHV-8; HHV-8-associated MCD), with the majority of cases consisting of human immunodeficiency virus (HIV) infected or immunocompromised patients [4]. Little is known about the etiology of HHV-8-negative MCD, referred to as idiopathic MCD (iMCD). iMCD accounts for up to 50% of all MCD cases and can lead to cytokine storm-mediated multiple organ system dysfunction, apart from other severe clinical manifestations [4].

The diagnosis of iMCD can be challenging because it is both a rare disorder and a mimicker of other clinical conditions such as malignant lymphoma, cancer, or autoimmune diseases [5]. Recently, Fajgenbaum et al. established evidence-based consensus diagnostic criteria for HHV-8-negative/iMDC, which are subdivided in major, minor, and exclusion criteria [4]. Major criteria concise enlarged lymph nodes and histopathologic features consistent with the iMCD spectrum (i.e., regressed/atrophic/atretic/hyperplastic germinal centers (GC), follicular dendritic cell (FDC) prominence, and vascularity). Minor criteria consist of 6 laboratory and 5 clinical features (elevated ESR or CRP, anemia, thrombocytopenia/cytosis, renal dysfunction or proteinuria, polyclonal hypergammaglobulinemia, hypoalbuminemia and constitutional symptoms, large spleen and/or liver, fluid accumulation, eruptive cherry angiomata or violaceous papules, lymphocytic interstitial pneumonitis, respectively) of which at least 2 must match the patient's symptoms (with ≥1 laboratory criterion). iMCD can be further subclassified into iMCD-thrombocytopenia, ascites, reticulin fibrosis, renal dysfunction, organomegaly (iMCD-TAFRO), or iMCD, not otherwise specified (iMCD-NOS) [2]. Considering exclusion criteria, it is crucial to rule out malignant, infectious, and autoimmune conditions that can mimic iMCD [4].

While therapeutic strategies for UCD are more or less complete surgical removal, the multicentric form of the disease involves many more different approaches. Considering the guidance from van Rhee et al. [6], the consensus treatment algorithm for iMCD patients, regardless of clinical severity, starts with administering anti-IL6-directed treatment [6]. Up to now, siltuximab, an anti-IL6 chimeric monoclonal antibody, is the only approved drug for the treatment of HHV-8-negative iMDC in Europe and the USA (US Food and Drug Administration, 2014; European Medicines Agency, 2016) [7, 8]. For people who do not respond to siltuximab, a broad spectrum of other treatment options exist, like tocilizumab or rituximab-based therapeutic approaches, to name a few; however, it is currently difficult to clearly determine their effectiveness [3].

## 2. Report of a Case

We report on a 78-year-old female patient diagnosed with HIV-negative, HHV-8-negative iMCD in 2021, with a history of nicotine abuse and comorbidities such as hyperthyroidism, coronary sclerosis, and bilateral knee gonarthrosis, leading to right total knee replacement in November 2020. As permanent therapeutic intervention, the patient received Thyronajod (175 *µ*g qd), pantroprazol (40 mg qd), Torasemid (5 mg qd), and Oxycodone (20 mg qd). During 2021, she received anti-SARS-CoV-2 vaccination (1st dose in April; 2nd dose in July).

As of November 2019, the patient was hospitalized due to progressive anemia (haemoglobin 10 g/dl, MCV 82 fl), persistent elevated levels of CRP (8–11 mg/dl), and weight loss (20 kg). Initially suspected as lymphoma, this diagnosis could not be clearly confirmed. CT scan of the thorax and abdomen revealed highly suspicious paraaortic and interaortocaval lymph nodes (largest paraaortic node: 3.3 × 2.6 × 1.1 cm). While subsequent examinations in March 2020 and March 2021 showed essentially steady lymph node progression (axillary lymph nodes from 6 to 8 mm; largest para-aortal node: 2.5 × 2.2 × .0 cm), these findings were considered not indicative for lymphoma.

The general condition of the patient appeared to be mainly stable since November 2019; Eastern Cooperative Oncology Group (ECOG) performance status has been assessed of 1 to 2.

However, as of June 2021, the patient was referred to our hospital by her general practitioner still with lymphoma as an initial tentative diagnosis. As well, she suffered from rheumatism-like musculoskeletal pain, mainly in the upper extremities.

**Figure 1:**
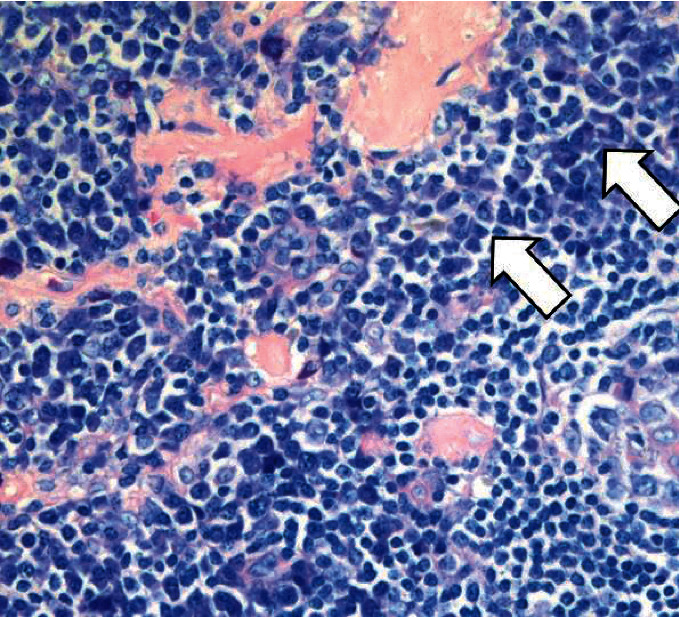
Histopathology. Involuted follicle (black arrow) with indistinct lymphocytic mantle and abundant perifollicular plasma cells (white arrows) (consistent with the plasma cell rich type of M Castleman).

Biopsy of a left-sided suprainguinal lymph node revealed atrophic germinal centers, positivity for CD21 in follicular dendritic cells, and a partial loss of proliferating B cells with abundant surrounding T-cells (Figure 1). Plasma cells were increased and appeared widely IgG-positive, exhibiting a polyclonal pattern for kappa and lambda light chains without enhanced expression of IgG4.

Both progressive lymphadenopathy in combination with histopathologic lymph node features are consistent with the iMCD spectrum (plasma cell subtype) and are referred to as major criteria.

Bone marrow aspirate studies including immunophenotyping of plasma cells showed a normal karyotype (46, XX) and could exclude myeloma due to low antigen expression (0.3%). Physical examination revealed splenomegaly, hypoalbuminemia (<35 g/l [32, 1 g/l]), pronounced increased soluble interleukin 2 receptor (sIL-2R) (1602 kU/l, normal 158–623), and highly elevated interleukin-6 (IL6) levels (64.1 pg/ml, normal <7.0 pg/ml). Further laboratory analyses were negative for HIV (serological testing), Epstein-Barr virus (EBV) (serological PCR and immunohistochemical testing), antinuclear antibodies (ANA), and HHV-8 infection (PCR testing). Vascular endothelial growth factor (VEGF) and interleukin-10 (IL) appeared in a normal range.

Since anemia, elevated CRP and IL6 levels, splenomegaly, and hypoalbuminemia meet 5 out of 11 minor consensus criteria, iMCD in the patient could be confirmed. The lack of thrombocytopenia, ascites, reticulin fibrosis, and renal dysfunction could clearly rule out iMCD-TAFRO, therefore we claimed the criteria are met for the existence of a less aggressive subtype iMCD-NOS.

Considering the consensus treatment algorithm for iMCD-NOS, the patient started therapy with siltuximab in August 2021 at a dose of 11 mg/kg intravenously every 3 weeks [6].

After 5 cycles of siltuximab, she presented in a general good condition with just a slight increase of lower limb edema. The therapy was well tolerated and no serious adverse side events could have been observed. Moreover, the patient experienced a remarkable decrease of rheumatism-like musculoskeletal pain. Haemoglobin as well as CRP and ferritin levels normalized (12.5 g/dl, normal 11.5–15.4 g/dl; 0.06 mg/dl, normal <0.50 mg/dl; 120.0 ng/ml, normal 13.0–150.0 ng/ml). TSH values decreased to 0.186 mU/l (normal <0.270–4.200 mU/l) while and FT4 values increased to 2.13 ng/dl (normal 0.90–1.70 ng/dl), both indicating hyperthyroidism which is an already known comorbidity in the patient.

CT scan of the thorax and abdomen revealed no essential changes in size of the spleen as well as of para-aortal and interaortocaval lymph nodes.

## 3. Discussion

More than half a century after Benjamin Castleman's initial description of a case, later termed as CD, a variety of lymphoproliferative disorders constitute the spectrum of CD. However, CD is a very rare and potentially serious lymphoproliferative disorder. International efforts have been made to define diagnostic criteria allowing to differentiate between particular forms of CD and to rule out mimickers of CD [4].

The patient who attended our hospital was in her eighth decade of life and had a history of nicotine abuse and comorbidities. Her clinical presentation with features like progressive anemia and persistent elevated levels of CRP did not automatically trigger the suspicion of CD since CD is a rare condition with an estimated 10-year period prevalence of 2.5 cases per million in North America, which may also be applicable to Germany [9]. After ruling out lymphoma as an initial tentative diagnosis, we examined the possibility for CD. Because of the wide range of potential clinical outcomes, a correct diagnosis of not only CD but also the subtype is essential for treatment strategy [10]. Further, thorough differential diagnostic examination following the scheme of Fajgenbaum et al. [4] could reveal the MCD-subtype HHV-8-negative iMCD-NOS in our patient and rule out lymphoma as the initial tentative diagnosis.

Anti-IL6-directed therapy is recommended for the treatment of HHV-8-negative iMDC [7, 8]. Since siltuximab is an anti-IL6 chimeric monoclonal antibody, we decided to treat the patient with this therapeutic agent following established guidelines [6].

Therapeutic outcome after 5 cycles of siltuximab administration (3 weeks each cycle) can be considered very promising. The patient reported toleration of the treatment and decrease of rheumatism-like musculoskeletal pain. IL6 plays a role in rheumatoid arthritis and it is a common observation that inhibiting IL6 ameliorates the symptoms of both rheumatoid arthritis and iMCD [11, 12]. Therefore, a positive effect on rheumatism-like musculoskeletal pain of our patient was not unexpected.

Blood values improved generally, in particular haemoglobin as well as CRP and ferritin levels normalized. CT scan of the thorax and abdomen exhibited stable disease. However, a slight increase of lower limb edema could have been observed as well as a slight deterioration of indicators for hyperthyroidism. Overall, siltuximab appears to be a safe and effective treatment of HHV-8-negative iMDC in our patient.

## 4. Conclusions

Improving iMCD patient outcomes is essentially dependent on timely correct diagnosis and sufficient therapy of the disease. The consistent use of international, evidence-based consensus diagnostic criteria described by Fajgenbaum et al. [4] as well as consensus treatment strategies [3] helped us rule out lymphoma as an initial tentative diagnosis and to minimize unfavourable outcomes for an iMCD-NOS patient. The application of anti-IL6-directed therapy with siltuximab appeared as a safe and effective treatment strategy.

## Data Availability

All data generated or analysed during this study are included in this published article.

## Abbreviations

ANA: Antinuclear antibodies
CD: Castleman disease
CT: Computed tomography
HHV-8: Human herpesvirus-8
HIV: Human immunodeficiency virus
IL: Interleukin
iMCD: idiopathic multicentric Castleman disease
TAFRO: Thrombocytopenia, ascites, reticulin fibrosis, renal dysfunction, organomegaly
UCD: Unicentric Castleman disease
VEGF: Vascular endothelial growth factor.
